# LEVERSC: Cross-Platform Scriptable Multichannel 3-D Visualization for Fluorescence Microscopy Images

**DOI:** 10.3389/fbinf.2022.740078

**Published:** 2022-03-17

**Authors:** Mark Winter, Andrew R. Cohen

**Affiliations:** Department of Computer Engineering, Drexel University, Philadelphia, PA, United States

**Keywords:** visualization, microscopy, light, computational analysis, biological microscopy, scripted rendering

## Abstract

We describe a new open-source program called LEVERSC to address the challenges of visualizing the multi-channel 3-D images prevalent in biological microscopy. LEVERSC uses a custom WebGL hardware-accelerated raycasting engine unique in its combination of rendering quality and performance, particularly for multi-channel data. Key features include platform independence, quantitative visualization through interactive voxel localization, and reproducible dynamic visualization *via* the scripting interface. LEVERSC is fully scriptable and interactive, and works with MATLAB, Python and Java/ImageJ.

## Introduction

Image analysis pipelines for 3-D fluorescence microscopy generally include image capture, image processing for object detection, tracking for time-lapse, object classification and ultimately statistical analysis of the extracted objects (Wait, 2014; Winter et al., 2016). Visualization at each of these stages is required to establish trust that the processing pipeline is capturing a true and meaningful summary of the data (Cohen, 2014). Three key requirements for effective visualization of 3-D multichannel images include 1) platform independence, 2) quantitative visualization, and 3) reproducible dynamic visualization. No existing image visualization tool satisfies this combination of requirements (Schroeder et al., 2006; Yushkevich et al., 2006; Meyer-Spradow et al., 2009; de Chaumont et al., 2012; Royer et al., 2015; Fantham and Kaminski, 2017; Gunther et al., 2019; O’Shaughnessy et al., 2019; Schmid et al., 2019; Jonsson et al., 2020; Pettersen et al., 2021; Napari Contributors, 2019). The LEVERSC visualization tool presented here was developed specifically to satisfy these requirements.

Platform independence means supporting the operating systems and common processing tools used for image analysis. Operating systems include Mac OS, Windows and Linux. Operating system independence for 3-D visualization is complicated by platform-specific support for hardware acceleration. For example, Mac OS has recently deprecated the OpenGL and OpenCL libraries that are widely used for 3-D visualization (Schroeder et al., 2006; Royer et al., 2015; Napari Contributors, 2019). Widely used tools for working with multi-channel 3-D fluorescence microscopy images include ImageJ, Python, and MATLAB as well as Knime and Julia. The goal of LEVERSC is to provide simple integration with any extensible environment, and to be used as easily as existing 2-D visualization on each platform. Because it is integrated in standard tools, LEVERSC works alongside fast image processing libraries such as the Hydra image processing library to provide intuitive visual feedback when designing image processing pipelines (Wait et al., 2019). LEVERSC is built using WebGL for 3-D rendering and NodeJS for the backend, making it lightweight, portable, and future proof.

**FIGURE 1:**
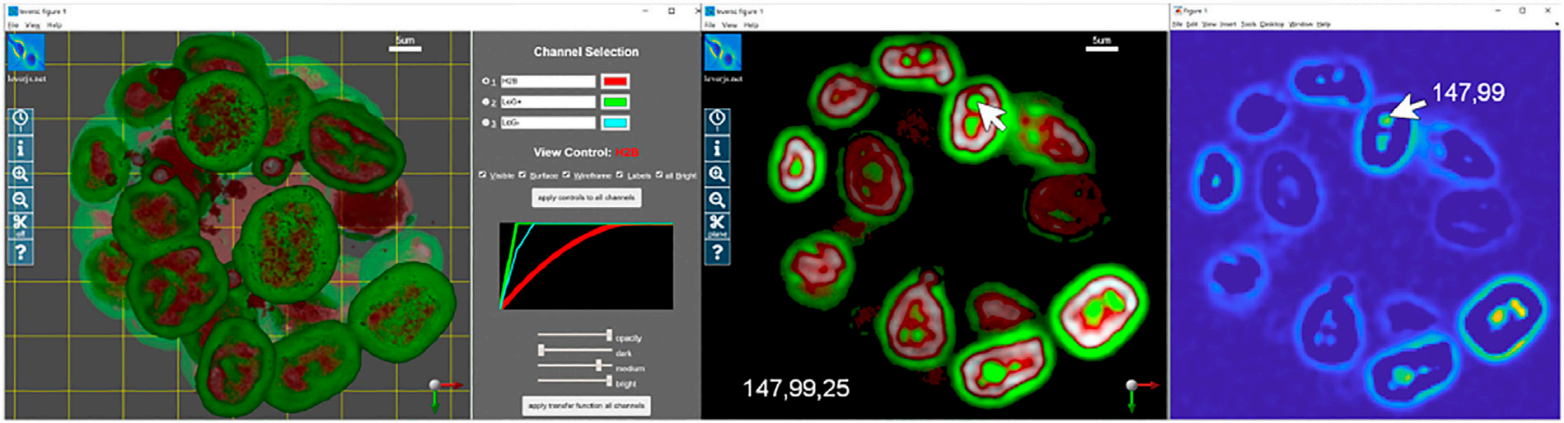
LEVERSC makes quantitative visualization at each step of the image processing pipeline as easy for 3-D multi-channel images as it is for 2-D images. The realistic rendering is enabled by a real-time anisotropic raycasting with perspective projection. The raw image and two computed images are visualized as a full stack (left), or as a slice at arbitrary orientation through the volume (center). Quantitative visualization recovers the precise 3-D voxel location, shown here with a 2-D single channel image view at *Z* location 25 (right).

Quantitative visualization means that the coordinates of any voxel on any channel can be precisely located. This is the location of the voxel within the raw image stack. High-quality 3-D visualization uses perspective projections that alter the spatial characteristics of the rectangular image volume to emulate the perspective vanishing point for 3-D structures on a 2-D screen. Quantitative visualization requires the ability to specify a 3-D location when viewing on a 2-D screen. Quantitative visualization is implemented in LEVERSC with the use of a view-aligned sampling plane. Figure 1 shows an example image showing nuclei of MCF10A cells labelled with H2B (histone 2B) in a 3-D spheroid (Ender et al., 2021; Gagliardi et al., 2021). The Laplacian of Gaussian filter is commonly used for nuclei detection (Wait et al., 2019). The positive and negative filter responses are extracted to separate image channels, and the volume is visualized (Figure 1, left). The sampling plane in the left panel is visible as a yellow grid, with shading of the volume changing behind the plane. The sampling plane can be set at arbitrary depth relative to the current view orientation. The sampling plane can be shown within the full volume (Figure 1, left), it can show only data behind it (clipped view), or it can show only data that intersects it (slice view), capturing a single slice through the volume (Figure 1, middle). The sampling plane, in combination with the mouse location, is used to generate the 3-D coordinate of a voxel location in the raw image stack (Figure 1, middle) that can then be viewed using a single channel 2-D image viewer from any processing environment (Figure 1, right).

Reproducible dynamic visualization means that all view parameters can be read into a scripting environment and written back into the visualization program. This allows view settings to be saved for exact reproducibility. This also allows the visualization to be animated. Image capture of the rendered image to the scripting environment is also critical so that animations can be saved as movies. Supplementary Movie S1 is an animation generated from the sample H2B image shown in Figure 1, generated as described in the online methods section.

Existing 3-D tools provide a variety of options for visualization of 3-D biological data. Visualization libraries such as the Visualization Toolkit (VTK) are fast and provide significant flexibility in visualization, but they require users to write a significant amount of custom code in *each* of their binding languages, such as Python or JAVA in order to provide interactive volumetric visualization (Schroeder et al., 2006). Tools such as Voreen and Inviwo are very flexible 3-D visualization prototyping tools, providing a platform for experimenting with different types of rendering and building volumetric renderers (Meyer-Spradow et al., 2009; Jonsson et al., 2020). These tools are excellent for technical users, but are not designed for quickly visualizing 3-D image data from multiple image processing environments. Other packages such as Icy, ChimeraX, itkSNAP and ImageTank, are built as standalone tools or tool ecosystems containing image-processing operations and visualization. However, each package limits users to only the operations available within the tool or requires that they write a tool extension or plugin for their purposes or export results for import into another tool for further processing or visualization (Yushkevich et al., 2006; de Chaumont et al., 2012; O’Shaughnessy et al., 2019; Pettersen et al., 2021). Powerful visualization tools such as ClearVolume, SciView, and FPBioimage, use related architecture components to LEVERSC, but focus largely on ImageJ/FIJI support (Royer et al., 2015; Fantham and Kaminski, 2017; Gunther et al., 2019). Similarly, 3DScript is an excellent ImageJ plugin for scripting movies using English commands, but does not support movie making in other image processing environments like Python or MATLAB (Schmid et al., 2019).

LEVERSC was developed as part of a collection of software tools for analyzing live-cell microscopy images, called LEVERJS (short for Lineage Editing and Validation) (Winter et al., 2016). The design of LEVERSC was inspired by the imagesc command in MATLAB, which can be used to quickly display a 2-D array as an image, by automatically mapping the full range of the array to a color palette. The ease of use of this command during image processing tasks makes it a powerful debugging tool. LEVERSC strives to provide a similar ease of use and consistency, from multiple image processing environments, while also providing quantitative imaging and scripting controls. The LEVERSC architecture was also designed to be as simple as possible to integrate with a request application programming interface (API) that should be supported by any scripting language or processing environment.

## Methods

### Installation

Both the leversc app and at least one client integration must be installed. First, install the LEVERSC app for the desired operating system. Next, follow the instructions for integrating LEVERSC into the client of choice (ImageJ, Python, or MATLAB).

### App Installation

#### MacOS App Installation

• Download and run the MacOS installer using the link at https://leverjs.net/leversc#macos-app-install

#### Windows App Installation

• Download and run the Windows installer using the link at https://leverjs.net/leversc#windows-app-install

#### Linux App Manual Installation

• Download the Linux Appimage using the link at https://leverjs.net/leversc#linux-app-manual-install

• Symlink the appimage file to a folder in $PATH (e.g., ∼/.local/bin).

ln -fs /path/to/leverjs-*.AppImage ∼/.local/bin/leverjs

### Client Integration

#### ImageJ Plugin

1. First, install the leversc app (see previous section).
2. Download the ImageJ plugin using the link at https://leverjs.net/leversc#imagej-plugin.
3. Copy the plugin jar file to the *plugins/3D* folder in your ImageJ executable directory.
4. The plugin will appear in the ImageJ Plugins menu as *Plugins->3D->Leversc Viewer*

Note: If using Fiji (highly recommended) then you may wish to create the 3D subfolder in plugins and place the jar file within, as it can make it easier to find. Alternatively, the jar file can be placed directly in the plugins folder and will be listed in the Fiji menu as Plugins->Leversc Viewer.

Note: There is currently no scripting support available from within Fiji/ImageJ. In order to control the viewer programmatically you need to use MATLAB or Python as described below.

#### Python Module–Requires Python 3.6 or Later

1. First, install the leversc app (see previous section).
2. From the command line:
  1. (Windows) ‘py -m pip install leversc’
  2. (Mac/Linux) ‘python3 -m pip install leversc’
  3. To validate install, start python, then:
  >>> import leversc; leversc.test_random()
3. For the full sample code, download the LEVERSC source directory (https://git-bioimage.coe.drexel.edu/opensource/leversc).

#### MATLAB Class–Requires MATLAB 2019B or Later

1. First, install the leversc app (see previous section).
2. Download the LEVERSC source directory (https://leverjs.net/leversc).
3. Extract the downloaded source and support folders to a convenient location.
4. Add the *src/MATLAB* subfolder to your MATLAB path, for example, by adding the statement *addpath('path/to/extracted/folder')* to your *startup.m* file.

Additional installation details can be found at: https://leverjs.net/leversc.

### Architecture

The LEVERSC visualization tool is a NodeJS application for visualization of multichannel 3-D volumetric data using WebGL. LEVERSC uses a local HTTP server port binding to communicate with image processing tools. Currently LEVERSC has plugins for ImageJ, Python and MATLAB. Additional plugins for KNIME and Julia are planned.

The LEVERSC HTTP request application programming interface (API) is designed to be simple to implement, so that image processing tools or scripting languages can be quickly extended to communicate with the LEVERSC viewer. The API can be implemented piecemeal, only requiring the image send request to be implemented in the simplest case. Additional API requests that control the visualization and allow scripted movie-making, can be implemented, but are not required for data visualization.

This architecture is very flexible and supports fast, cross-platform communication between any environment that supports HTTP POST/GET requests. A detailed listing of all API requests is available in the online documentation linked from the main LEVERSC repository readme at: https://leverjs.net/leversc.

### Raycast Renderer

LEVERSC uses a raycast sampler implemented in a WebGL fragment shader. For each pixel in the display a view ray is cast outward through the image volume. The image voxel values are sampled uniformly along the ray corresponding with the size of image voxels. At each sample a user-defined transfer function, detailed in the subsequent section, is applied to convert from normalized voxel values to emissive intensities and opacity. As sampling continues along each ray, color intensity and opacity (alpha) are accumulated using standard alpha blending calculations. Once all ray sampling is completed, the blended color and opacity are composited with the render background to draw the final rendered frame to the screen WebGL window.

The LEVERSC visualization tool also supports three clip modes: off, front, and plane. These clipping modes control the rendering at and behind the user-controlled view-aligned clipping plane. With clipping turned off, the full depth of the volume is always rendered. Front clipping will render only the portion of the volume behind the clipping plane. Plane clipping will render only the image data that directly intersects the clipping plane, allowing users to view image slices at arbitrary orientation. The 3-D image coordinates of the mouse pointer projected onto the clipping plane is always displayed in the bottom left of the LEVERSC window so that users can easily determine exact voxel coordinates when using LEVERSC to design or debug image processing algorithms.

### Transfer Function

The transfer function is a per-channel parameterized function that maps voxel values (normalized on [0,1]) to emissive intensity values (on [0,1]) for modeling light-transport in the ray caster. The function is a monotonic quadratic. In the user interface, the function is determined by 3 sliders, dark, medium, and bright. The “dark” slider defines the largest voxel value that is completely transparent, all values at or below the “dark” level are mapped to 0 and are fully transparent. The “bright” slider defines the smallest voxel value that is fully emissive and opaque, all values at “bright” or above are fully opaque and emissive. The “medium” slider is the output value in [0,1] of the midpoint value between “dark” and “bright”, in essence defining the curvature of the quadratic, if “medium” is larger than 0.5 the quadratic will be concave down, if “medium” is less than 0.5 the quadratic will be concave up, and if “medium” is 0.5 then the function will be linear. Below the transfer function equation and parameters is shown for a single channel, mapping from the input voxel intensity $i_{in}$ to output intensity $i_{out}$ ,

$$i_{out}=\{\begin{array}{ll} ai_{in}^{2}+bi_{in}+c, & i_{in}\in \left[i_{dark},i_{bright}\right]\\ 1, & i_{in}\in \left[i_{bright},1\right]\\ 0, & i_{in}\in \left[0,i_{dark}\right] \end{array}$$

The parameters a,b,c are chosen such that $i_{out}\in \left[0,1\right]$ for $i_{in}\in \left[i_{dark},i_{bright}\right]$ and $i_{out}=i_{mid}$ when $i_{in}=\left(i_{dark}+i_{bright}\right)/2$ . The parameters are also constrained such that $i_{out}$ increases monotonically over the input range. The transfer function mapping, with per-channel parameters, is implemented in the WebGL raycasting fragment shader. This allows the user to adjust the transfer function settings and have the visualization update in real-time. Each channel also has a globally adjustable opacity (alpha). Once a ray is fully opaque or the ray has reached a far edge of the volume data, the total accumulated color is rendered to the screen, providing real-time volumetric rendering.

## Results

**FIGURE 2:**
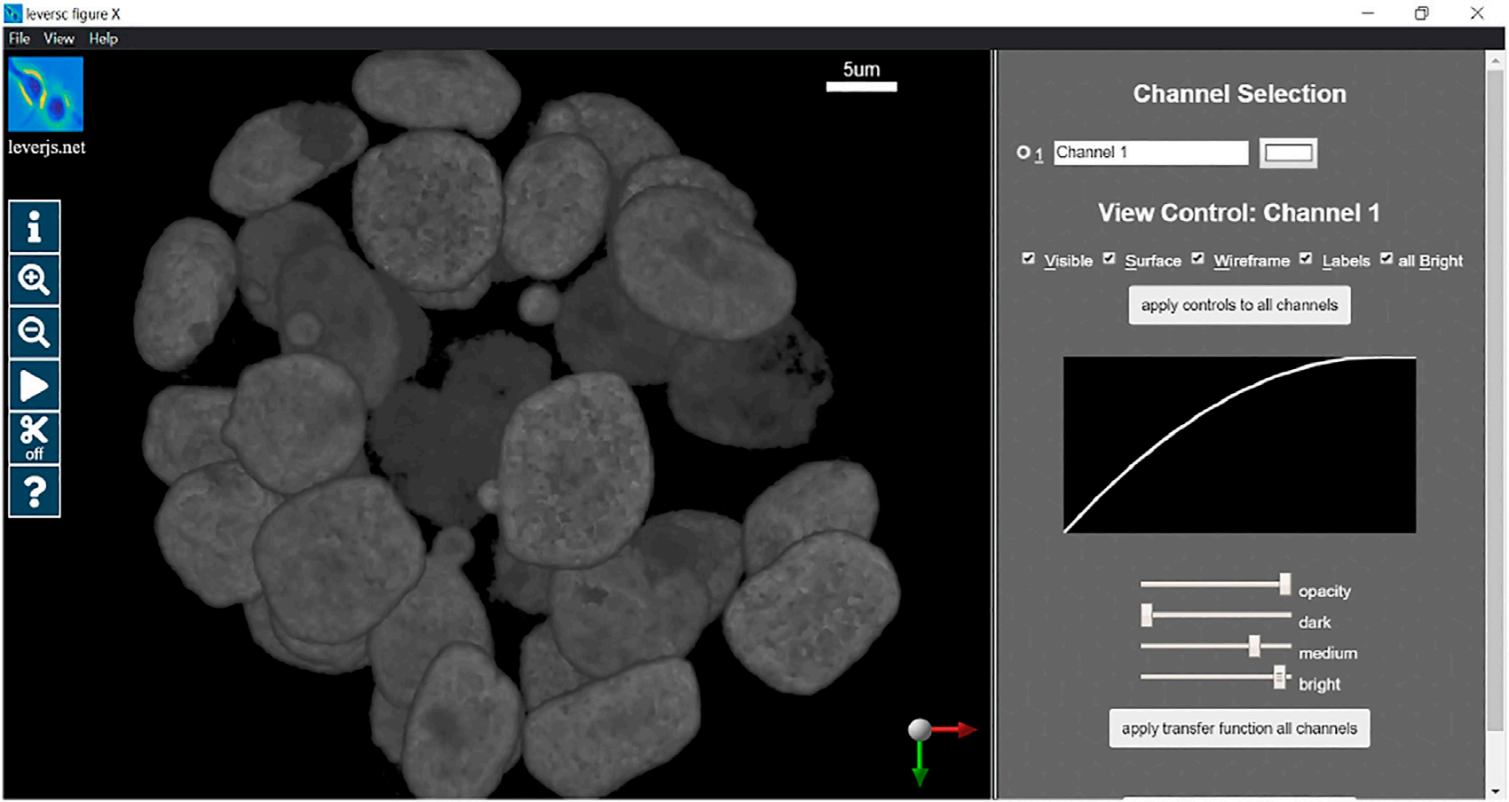
LEVERSC render parameter selection interface. Colors, alpha blending and intensity mapping transfer functions are all fully controllable *via* the user interface and the API.

In this section we will provide a detailed example of usage of the LEVERSC tool. This example shows the use of many of the LEVERSC application programming interface commands to create a high-quality rendered movie using the MATLAB scripting language. The full example script source code (*sampleVolumeMovie.m*) is available in the *src/MATLAB* directory of the LEVERSC repository. Figure 2 details the rendering interface used to control and indicate image coloring, alpha values and the intensity mapping transfer functions. The MP4 movie resulting from following this example script is included as Supplementary Movie S1.

We begin by loading some image data, in this case from the sample.LEVER file distributed along with the LEVERSC repository. Any format image readable by the scripting environment can be substituted.


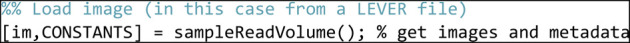


A leversc class object must be initialized, here we initialize the leversc class with image data and metadata (metadata fields such as PixelPhysicalSize are important for correct data visualization).


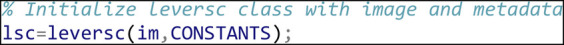


A reproducible movie render should set the rendering parameters consistently at the beginning of rendering to properly visualize the data. In this case we use the LEVERSC tool interface to interactively identify good visualization values, then use the /renderParams API call to read the current settings. The rendering parameters are set interactively *via* the UI as shown in Figure 2.

Once the rendering parameters are set, they are read back into MATLAB and hardcoded for subsequent reuse: 
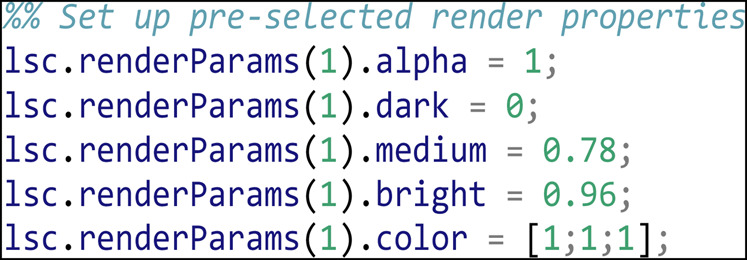


We disable the display of most UI elements


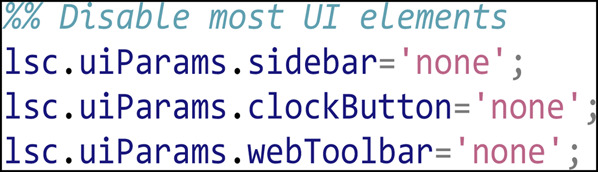


We reset the view parameters to defaults for the start of the movie:


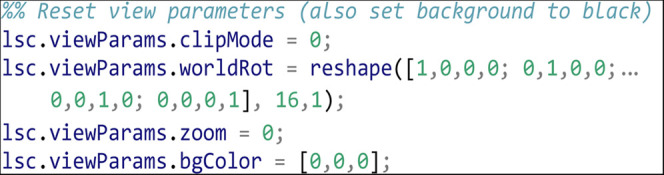


The first step in this movie is to apply a quick animated zoom to fill the display with the actual data in the volume, capturing frames for each zoom level. Since our movie will run at 10 frames per second (fps) we interpolate our zoom over 10 frames (a 1 s zoom):


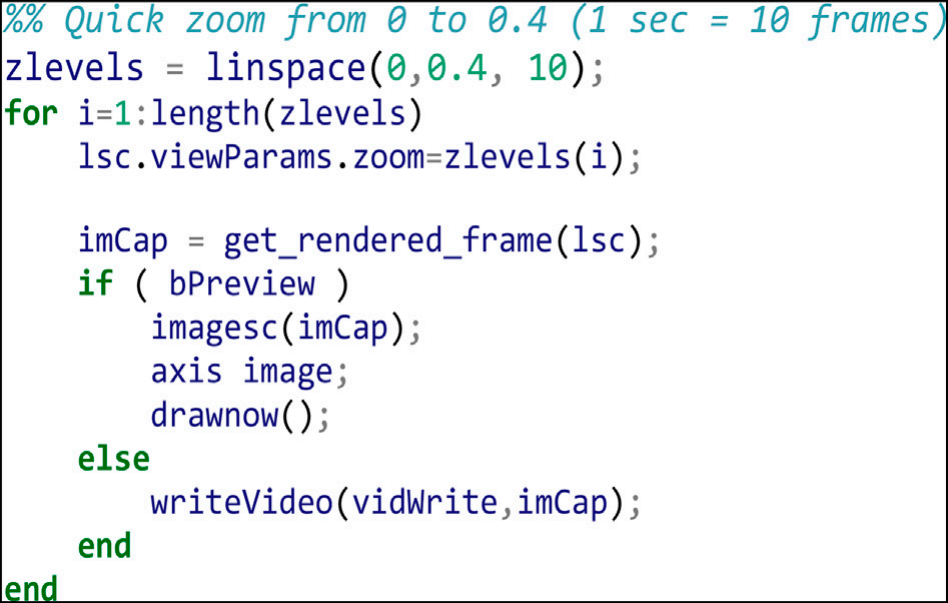


Apply a 5 s (50 frame) rotation of 180 degrees about the y-axis:


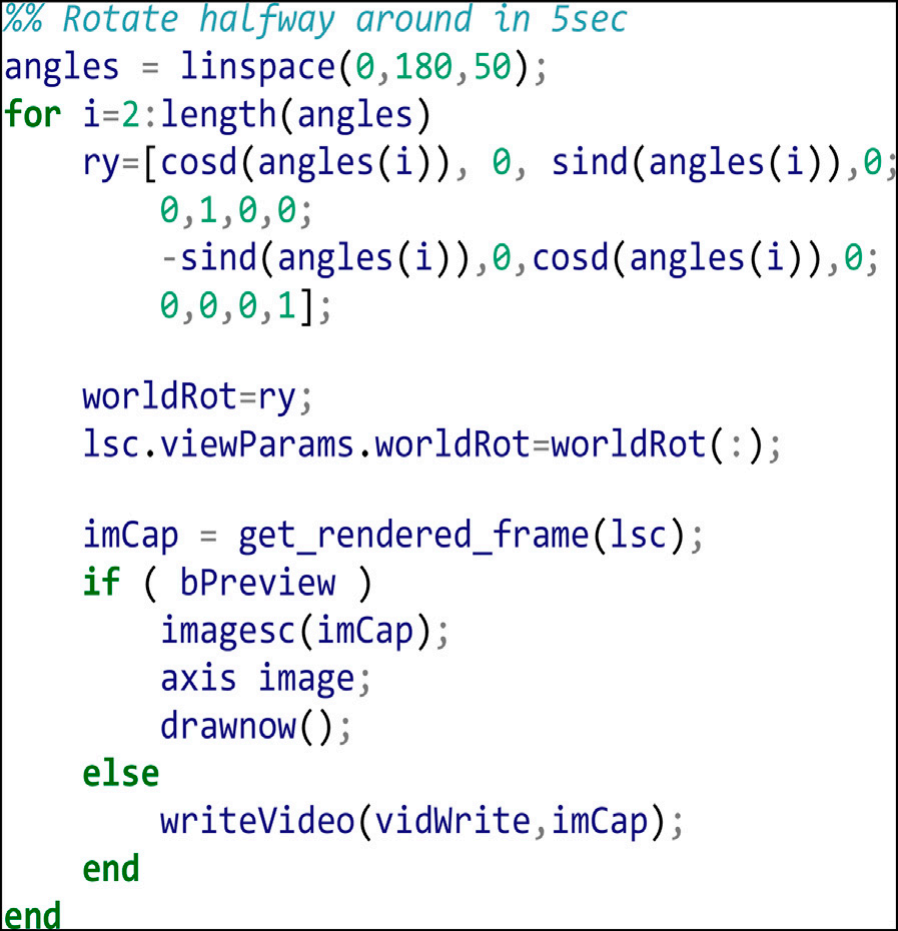


Move the sampling plane to the edge of the volume and turn on planar clipping. Then we animate moving the plane to just a little back from the volume center. The plane animation is 2 s (20 frames) long:


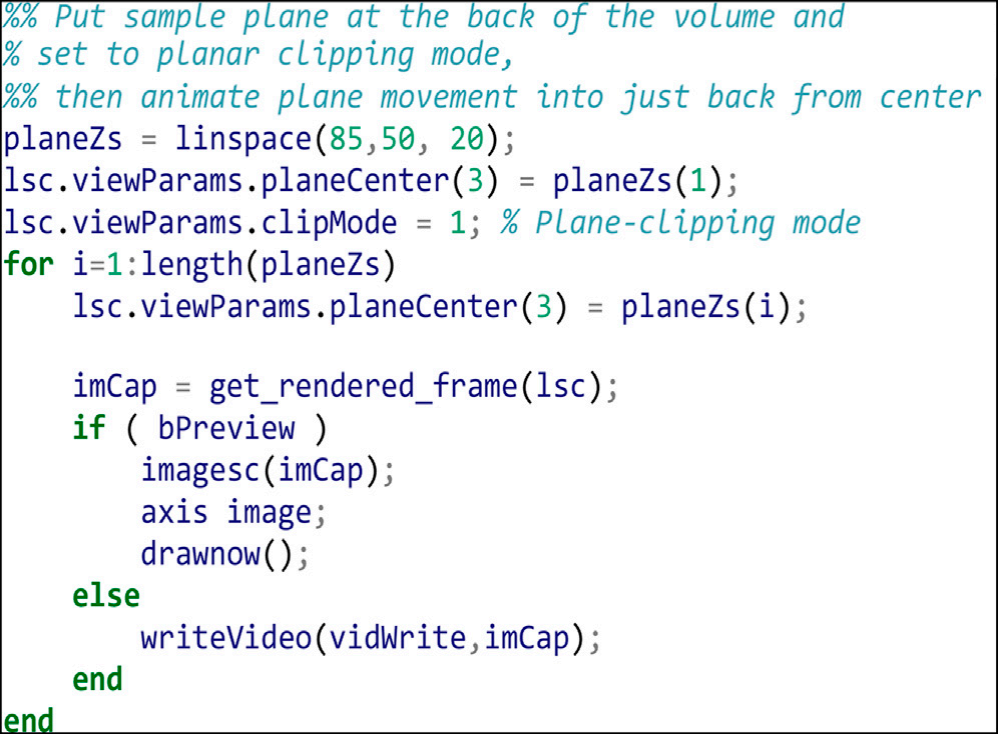


Apply another 180-degree rotation to rotate the volume the rest of the way back to the starting view. This time we show matrix multiplication for the world rotation matrix by a delta rotation matrix:


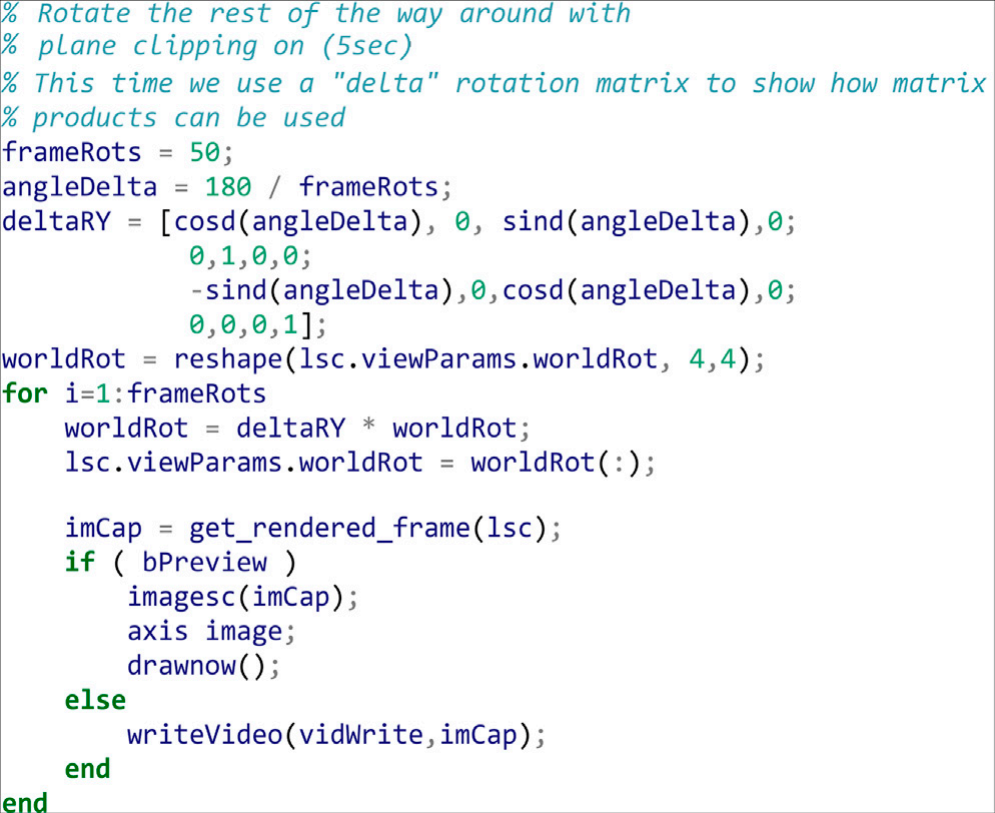


As a final animation, we change the plane clipping mode to slice sampling, and animate moving out of the volume towards the camera:


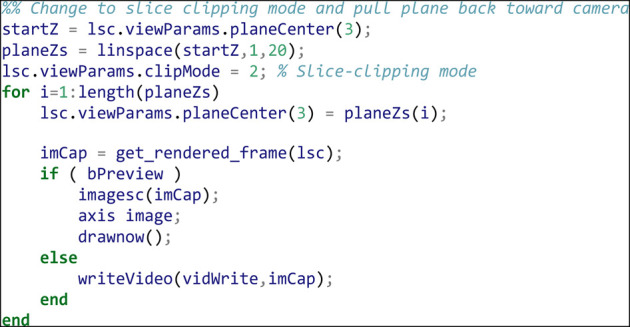


Using the example shown here, as well as the full viewParams and renderParams API calls, complex camera and movie effects can be built through interpolation of multiple parameters at each frame. Supplementary Movie S1 shows the full results of running the code fragments above.

## Discussion

Effective analysis of complex anisotropic image data requires effective visualization at every step of the processing pipeline. Visualization needs to be available from any platform, operating system and processing environment. It should be as easy to use as inbuilt 2-D image visualization. Visualization also needs to be quantitative–able to precisely identify the image stack location of every voxel at any view orientation and scale. Finally, the visualization needs to be fully controllable from any scripting environment, allowing view settings to be recorded for subsequent reproducibility, and view settings to be played back for generating movies. The LEVERSC visualization tool described here addresses all these requirements.

LEVERSC is designed to be useful to a broad audience of microscopists, biologists, and image analysists. However, the focus on compatibility and fast, interactive rendering does come with trade-offs. WebGL provides a highly compatible rendering interface with built-in support for fast 3-D image sampling. However, the use of a WebGL GPU texture sampler requires that the image volume be quantized to 8-bits per channel. This also requires that the entire image volume fit in GPU memory, and the LEVERSC tool cannot display images larger than the GPU memory available. However, users can still quickly crop a region of interest from a large volume to be visualized in LEVERSC, or they may downsample the volume in their image processing environment to view an approximation of the volume. We are also investigating multiresolution progressive rendering support in LEVERSC. This is already supported in the LEVERJS microscope analysis tool, but that rendering requires preprocessing and use of a specialized image format. While it would also be ideal to sample and display images at their full bit depth, we believe the speed of GPU textures is worth this sacrifice, particularly since most monitor displays generally have bit depth of 8–10-bits per color, and the current LEVERSC plugins use linear normalization to minimize the quantization error.

The architecture of LEVERSC makes it highly platform independent and compatible with other tools since it runs as a separate NodeJS process using standard HTTP requests for communication. However, this does increase the memory requirements for visualization as the image volume must be sent (copied) from the originating process to the LEVERSC visualization process. We have not found this to be an issue with modern hardware and modern live-cell microscope images, but could certainly be a concern with large electron microscopy (EM) data.

LEVERSC is a lightweight high-performance tool that provides a high-quality rendering *via* true raycasting using per voxel compositing across all image channels. It runs on all three major operating systems and is usable from ImageJ, Python and MATLAB. LEVERSC will support Knime using the ImageJ plugin architecture, with support for the Julia scripting language coming soon. The LEVERSC sampling plane enables quantitative visualization by projecting 2-D pointer location, together with the arbitrary plane location into image coordinates. LEVERSC is fully scriptable, providing image capture as well as programmatically controllable view, render and user interface settings. LEVERSC is available free and open source (MIT license). Source code for the scripting client and download links for the visualization executable can be found at https://leverjs.net/leversc.

## Data Availability

The sample data provided in this study is included in the source repository, found at: https://leverjs.net/leversc.

## References

[B1] Cohen A. R. (2014). Extracting Meaning from Biological Imaging Data. Mol. Biol. Cel 25, 3470–3473. 10.1091/mbc.E14-04-0946 PMC4230605 25368423

[B2] de Chaumont F. Dallongeville S., Chenouard N. Hervé N., Pop S., Provoost T. (2012). Icy: an Open Bioimage Informatics Platform for Extended Reproducible Research. Nat. Methods 9, 690–696. 10.1038/nmeth.2075 22743774

[B3] Ender P. Gagliardi P. A. Dobrzyński M. Dessauges C. Höhener T. Jacques M.-A. (2021). Spatio-temporal Control of ERK Pulse Frequency Coordinates Fate Decisions during Mammary Acinar Morphogenesis. In review and bioRxi. 10.1101/2020.11.20.387167 36113484

[B4] Fantham M. Kaminski C. F. (2017). A New Online Tool for Visualization of Volumetric Data. Nat. Photon. 11, 69. 10.1038/nphoton.2016.273

[B5] Gagliardi P. A. Dobrzyński M. Jacques M.-A. Dessauges C. Ender P. Blum Y. (2021). Collective ERK/Akt Activity Waves Orchestrate Epithelial Homeostasis by Driving Apoptosis-Induced Survival. Dev. Cel. 56 (12), 1712–1726. 10.1016/j.devcel.2021.05.007 In Press 34081908

[B6] Gunther U. Pietzsch T. Gupta A. Harrington K. I. S. Tomancak P. Gumhold S. (2019). “Scenery: Flexible Virtual Reality Visualization on the Java VM”. In 2019 IEEE Visualization Conference (VIS) 1–5, Vancouver, BC, Canada, 20-25 Oct. 2019. IEEE. 10.1109/VISUAL.2019.8933605

[B7] Jonsson D. Steneteg P. Sunden E. Englund R. Kottravel S. Falk M. (2020). Inviwo — A Visualization System with Usage Abstraction Levels. IEEE Trans. Vis. Comput. Graph. 26, 3241–3254. 10.1109/TVCG.2019.2920639 31180858

[B8] Meyer-Spradow J. Ropinski T. Mensmann J. Hinrichs K. V. (2009). A Rapid-Prototyping Environment for Ray-Casting-Based Volume Visualizations. IEEE Comput. Graph. Appl. 29, 6–13. 10.1109/MCG.2009.130 24806774

[B14] Napari Contributors (2019). Napari: A Multi-Dimensional Image Viewer for Python. 10.5281/zenodo.3555620

[B9] O’Shaughnessy E. C. Stone O. J. LaFosse P. K. Azoitei M. L. Tsygankov D. Heddleston J. M. (2019). Software for Lattice Light-Sheet Imaging of FRET Biosensors, Illustrated with a New Rap1 Biosensor. J. Cel Biol. 218, 3153–3160. 10.1083/jcb.201903019 PMC6719445 31444239

[B10] Pettersen E. F. Goddard T. D. Huang C. C. Meng E. C. Couch G. S. Croll T. I. (2021). UCSF ChimeraX: Structure Visualization for Researchers, Educators, and Developers. Protein Sci. Publ. Protein Soc. 30, 70–82. 10.1002/pro.3943 PMC7737788 32881101

[B11] Royer L. A. Weigert M. Günther U. Maghelli N. Jug F. Sbalzarini I. F. (2015). ClearVolume: Open-Source Live 3D Visualization for Light-Sheet Microscopy. Nat. Methods 12, 480–481. 10.1038/nmeth.3372 26020498

[B12] Schmid B. Tripal P. Fraaß T. Kersten C. Ruder B. Grüneboom A. (2019). 3Dscript: Animating 3D/4D Microscopy Data Using a Natural-Language-Based Syntax. Nat. Methods 16, 278–280. 10.1038/s41592-019-0359-1 30886414

[B13] Schroeder W. Martin K. Lorensen B. (2006). The Visualization Toolkit: an Object-Oriented Approach to 3D Graphics ; [visualize Data in 3D - Medical, Engineering or Scientific ; Build Your Own Applications with C++, Tcl, Java or Python ; Includes Source Code for VTK (Supports Unix, Windows and Mac)]. Clifton Park, NY: Kitware, Inc.

[B15] Wait E. (2014). Visualization and Correction of Automated Segmentation, Tracking and Lineaging from 5-D Stem Cell Image Sequences. BMC Bioinformatics 15, 328. 10.1186/1471-2105-15-328 25281197 PMC4287543

[B16] Wait E. Winter M. Cohen A. R. (2019). Hydra Image Processor: 5-D GPU Image Analysis Library with MATLAB and Python Wrappers. Bioinforma. Oxf. Engl. 35 (24), 5393–5395. 10.1093/bioinformatics/btz523 PMC7904059 31240306

[B17] Winter M. Mankowski W. Wait E. Temple S. Cohen A. R. (2016). LEVER: Software Tools for Segmentation, Tracking and Lineaging of Proliferating Cells. Bioinformatics 32, 3530–3531. 10.1093/bioinformatics/btw406 27423896 PMC5181556

[B18] Yushkevich P. A. Piven J. Hazlett H. C. Smith R. G. Ho S. Gee J. C. (2006). User-guided 3D Active Contour Segmentation of Anatomical Structures: Significantly Improved Efficiency and Reliability. NeuroImage 31, 1116–1128. 10.1016/j.neuroimage.2006.01.015 16545965

